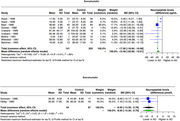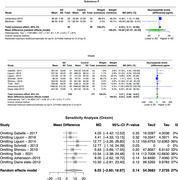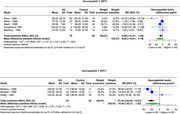# Neuropeptide Alterations in Alzheimer's Disease: A Meta‐Analysis of Cerebrospinal Fluid Concentrations

**DOI:** 10.1002/alz.090117

**Published:** 2025-01-09

**Authors:** Muneeb Ahmad Muneer, Vinay Suresh, Aman Milind Bhonsale, Sonit Sai Vasipalli, Vignesh Kumar, Poorvikha Gowda, Binish Javed, Mainak Bardhan, Ravindra Kumar Garg, Hardeep Singh Malhotra

**Affiliations:** ^1^ Research Peer Network ‐ Neurology Study Group, Lucknow, Uttar Pradesh India; ^2^ Allama Iqbal Medical College, Lahore, Punjab Pakistan; ^3^ King George's Medical University, Lucknow, Uttar Pradesh India; ^4^ All India Institute of Medical Sciences, Nagpur, Nagpur, Maharashtra India; ^5^ Jawaharlal Institute of Postgraduate Medical Education and Research, Puducherry, Puducherry India; ^6^ Lokmanya Tilak Municipal Medical College, Mumbai, Maharashtra India; ^7^ St John’s Medical College, Bangalore, Karnataka India; ^8^ Atal Bihari Vajpayee Institute of Medical Sciences and Dr. Ram Manohar Lohia Hospital, New Delhi, Delhi India; ^9^ Miami Cancer Institute, Baptist Health South Florida, USA, Miami, FL USA

## Abstract

**Background:**

Neuropeptides are crucial proteins in the central nervous system, which significantly influence neurophysiological processes. This analysis explores cerebrospinal fluid alterations in Alzheimer's disease, offering insights to better understand the condition and explore novel diagnostic and therapeutic avenues.

**Method:**

We systematically searched MEDLINE (PubMed), EMBASE, Cochrane, and Scopus using specific search strategies. Following PRISMA guidelines, our screening and extraction included studies that investigated neuropeptide concentrations in Alzheimer's patients' cerebrospinal fluid (CSF). Utilizing the 'meta' R package, particularly the 'metacont' tool, we analyzed mean concentration levels and standard differences via the random effects model. Data pooling utilized inverse variance weighting, with I² and tau² assessing heterogeneity. The restricted maximum−likelihood estimator and Q−profile informed tau² and its confidence interval. The primary outcome was the mean difference in CSF neuropeptide levels between Alzheimer's patients and controls, considering reported units separately.

**Result:**

In our analysis of neuropeptides across diverse studies involving Alzheimer's Disease (AD) patients and controls, we observed distinct neurochemical alterations. Somatostatin showed a significant mean difference of ‐15.48 pg/mL (95% CI: ‐20.95 to ‐10.01, I² = 89%) in 9 studies (141 AD, 203 controls), and ‐8.05 pmol/L difference (95% CI: ‐13.35 to ‐2.76, I² = 0%) in 2 studies (44 AD, 57 controls). Neuropeptide Y (NPY) showed ‐8.83 pg/mL difference (95% CI: ‐19.81 to 2.16, I² = 65%) in 5 studies (76 AD, 95 controls), and ‐16.85 pmol/L difference (95% CI: ‐35.21 to 1.52, I² = 71%) in 3 studies (87 AD, 62 controls). Orexin exhibited a difference of 8.03 pg/mL (8 studies: 235 AD, 177 controls; ‐2.60; 18.67, I² = 27%, p‐value: 0.14). After excluding a study that significantly reduced heterogeneity, the adjusted result was 12.77 (95% CI: 1.16 ‐ 24.39, p‐value: 0.03). Substance P also showed a reduction in mean difference by ‐16.52 pg/mL (2 studies: 45 AD, 35 controls; ‐67.47; 34.42, I² = 87%) but was not statistically significant.

**Conclusion:**

Somatostatin exhibited a consistent decrease in levels across studies, indicating potential relevance to AD pathophysiology. Additionally, orexin demonstrated a significant increase in levels in individuals diagnosed with Alzheimer's.